# Phase angle as a marker of outcome in hospitalized pediatric patients. A systematic review of the evidence (GRADE) with meta-analysis

**DOI:** 10.1007/s11154-023-09817-1

**Published:** 2023-07-24

**Authors:** Rocío Fernández-Jiménez, Rafael Martín-Masot, Isabel Cornejo-Pareja, Isabel M. Vegas-Aguilar, Marta Herrador-López, Francisco J. Tinahones, Víctor Manuel Navas-López, Diego Bellido-Guerrero, José Manuel García-Almeida

**Affiliations:** 1 Department of Endocrinology and Nutrition, Virgen de la Victoria Hospital (IBIMA), Malaga University, Campus Teatinos S/N 29010, Malaga, Spain; 2 Instituto de Investigación Biomédica de Málaga-Plataforma BIONAND (IBIMA), Virgen de la Victoria University Hospital, 29010 Málaga, Spain; 3 Centro de Investigación Biomédica en Red de la Fisiopatología de la Obesidad y Nutrición (CIBEROBN), Instituto de Salud Carlos III (ISCIII), 29010 Málaga, Spain; 4 Pediatric Gastroenterology and Nutrition Unit, Hospital Regional Universitario de Málaga, Málaga, Spain; 5 Department of Endocrinology and Nutrition, Complejo Hospitalario Universitario de Ferrol, La Coruña, Ferrol, Spain

**Keywords:** Mortality, Phase angle, Bioelectrical impedance, Length of stay, Long-term hospitalization, Quality of life

## Abstract

Phase angle (PhA) is a valuable tool for evaluating the nutritional and inflammatory status, which can accompany acute and severe disorders. PhA is a cellular health biomarker, whose value is particularly substantial due to the negative consequences of these situations in the pediatric population. Relevant literature was collected with the aim of comprehensively analysing the evidence on the association between an altered PhA can serve as a predictive-marker for mortality and poor-outcomes in at-risk-pediatric patients. Understanding this relationship could have significant implications for identifying high-risk individuals and implementing timely interventions. A systematic review with meta-analysis was conducted in the primary electronic databases from inception until January 2023. Overall, four studies with a total of 740 patients were eligible for our analysis. Evidence demonstrates that PhA is associated with nutritional status, reflecting undernutrition and changes in body composition related to illness. This review suggests that PhA can indeed be used as an indicator of nutritional status and a tool for predicting prognosis, including mortality and poor-outcomes, in hospitalized pediatric patients. A low PhA was associated with a significant mortality risk [RR:1.51;95%CI (1.22–1.88),p = 0.0002;I2 = 0%,(p = 0.99)] and an increased complications risk [OR:8.17;95%CI (2.44–27.4),p = 0.0007;I2 = 44%,(p = 0.18)]. These findings highlight the importance of taking a comprehensive approach to clinical nutrition, integrating multiple evaluation aspects to establish an accurate diagnosis and personalized therapeutic plans. While PhA emerges as a valuable tool for assessing the risk of malnutrition and as a prognostic-indicator for poor-outcomes in pediatric patients. Further future studies are needed to focus on investigating this relationship in larger and diverse population to strengthen the evidence base.

**Graphical Abstract:**

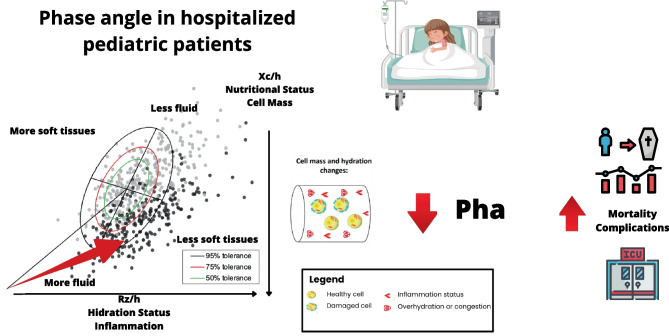

## Background

In clinical practice there is no single tool to assess the nutritional status of a patient. Traditionally, basic anthropometric parameters such as weight, height and body mass index (BMI) have been used for this. These parameters are essential and must continue to be taken into account, but they are not sensitive enough to assess the different components of weight or changes in body composition. In order to evaluate these changes, the need to incorporate new nutritional assessment parameters such as the Phase angle (PhA) is raised, which, although it still have limitations within clinical practice, may be more sensitive, specific and reproducible [1].

Bioelectrical impedance (BIA) is a simple, non-invasive method that, due to its ease of use and low cost, has been used in recent decades to estimate the body composition of healthy and sick subjects. Despite its widespread use in clinical nutrition, this technique does not directly measure body composition, and the lack of standardized methods, quality control procedures, and the fact that its accuracy depends on the formulas used makes this technique subject to possible biases [2].

**Fig. 1:**
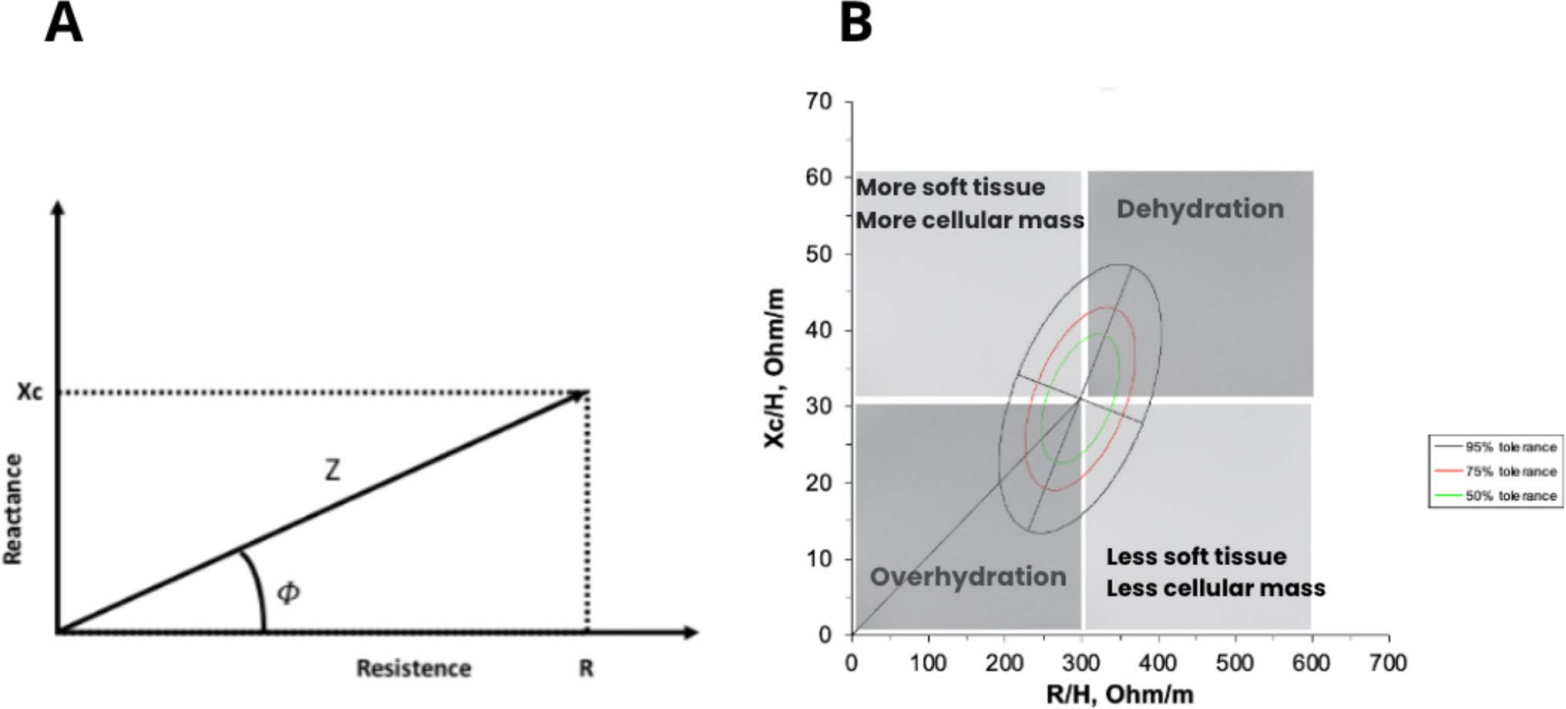
**A** Geometric relationships of impedance components. **B** RXc graph. The BIVA nomogram uses tolerance ellipses to plot reference values and assess the position of vectors. Values outside the 95th percentile are considered abnormal. The vector's position and length provide information about disease status and cell membrane function. A longer vector indicates high or low R values, indicating dehydration (quadrant 1) or overhydration (quadrant 3) respectively. Sideways migration of the vector due to high or low XC indicates an increase (quadrant 2) or decrease (quadrant 4) in dielectric mass of soft tissues. The interpretation remains the same when using Z scores. Quadrant divisions are indicative rather than strict distinctions. Xc = reactance; R = resistence; Φ = Phase angle; z = impedance; H = height (meters). References [8, 9]

Bioelectrical vector impedance (BIVA), developed by Piccoli et al. [3], allows to directly measure the body's opposition to alternating current, that is, the impedance (Z), which consists of two components: resistance (R) and reactance (Xc) [4]. Resistance is the opposition offered by the body to the flow of an alternating electrical current and is inversely related to the water and electrolyte content of the tissue. Reactance is related to the capacitance properties of the cell membrane, and variations may occur depending on its integrity, function, and composition [5]. These two parameters provide direct information on hydration status, body cell mass, and cell integrity, without algorithm-dependent errors and without requiring assumptions such as constant tissue hydration [4]. From the vector relationship between these two crude impedance parameters, the PhA can be calculated by means of the following formula: tan^−1^(Xc/R) × 180/π (Fig. 1A).

### Fundamentals and methodology for Phase angle measurement

The PhA is physiologically characterized as an index of the integrity and vitality of the cell membrane and expresses the quantity and quality of the soft tissues. PhA is positively correlated with lean body mass and cell mass, but is inversely related to the ratio of extracellular to intracellular fluid in healthy adults and children [6]. It is considered an indicator of cellular health, since high PhA values ​​indicate greater cellularity and integrity of the membrane and better cellular function [4, 7]. However, lower PhA values ​​are indicative of a worse prognosis and higher morbidity and mortality. Due to its direct relationship with the state of cellular health, this angle has been proposed as a prognostic marker in certain clinical conditions [2].

To obtain the PhA value, a distal tetrapolar, monofrequency and vectorial BIA equipment is used [8, 9]. To perform its measurement, 4 electrodes are placed on the right side of the body, two of them on the hand and two on the foot. It is important to comply with the measurement protocol, the standardized conditions prior to it, the position of the patient and the placement of the electrodes so that the electrical determinations are not affected [2].

This equipment uses a 50 kHz signal to directly measure R and Xc standardized for height, which can be plotted on the RXc graph. The vector that is produced (Z) has a length inversely related to total body water and the combination of the vector's length and its direction, defined as the PhA, is an indicator of hydration status [7]. The RXc plot (Fig. 1B) is a probability distribution that classifies a vector according to its distance from the mean healthy vector. The variability of the Z vector is represented in tolerance ellipses (50%, 75% and 95%). Vectors within the 50% tolerance ellipse are within normal hydration, while vector elongation to 75% and above this percentage and 95% indicates moderate and severe dehydration, respectively. On the contrary, the shortening of the vectors within these percentages in the reference ellipses in the lower range indicates an increasing fluid overload. Vectors to the left of the major axis reflect increasing cell mass and vectors to the right indicate decreasing cell mass, respectively.

### Phase angle and malnutrition in pediatric population. Predictor of mortality and complications

Malnutrition is very prevalent in critically ill children. Disease-related malnutrition is characterized by an early exchange of fluids from the intracellular water space to extracellular water with a concomitant decrease in cell mass. These disease-related alterations in fluid distribution are reflected by decreasing PhA [4].

Several studies in developing countries showed that malnutrition may affect 50% of children and adolescents admitted to the pediatric intensive care unit (PICU) [10], and in turn is associated with increased morbidity and mortality, including increased risk of infection due to transient immune disorders, inadequate wound healing, reduced bowel function, increased dependence on mechanical ventilation and longer hospital stays [1–3].

Body composition appears to be able to predict clinical prognosis, which is extremely important as early identification of severity allows anticipation of therapeutic measures that may be decisive in patient’s outcomes. Studies have shown that reduced muscle mass is an indirect risk factor for mortality and is associated with a longer length of hospital stay [11, 12].

There are several techniques to assess body composition, such as mid-upper arm circumference and triceps skinfold thickness[13, 14] that describe fat deposits and lean mass [15], but the most complete technique and the one that gives us more values regarding body composition is the bioelectrical impedance analysis.

PhA is a cellular health marker, that detects malnutrition and inflammation that can accompany acute and/or severe pathologies, it has has been used in recent years as an evaluation technique with a prognostic factor for mortality or complications in different pathologies [16–21] like cancer [22–26], liver diseases [27–29], kidney diseases renal [30, 31] and critically ill patients [32–34]. In addition, there are strong indications of an association between decreased PA values ​​and mortality [35]. One of the most studied pathologies is cancer, where it is observed that the PhA value can detect a higher or lower survival during treatment and in the evolution of the disease depending on the cellular status of the patient [36, 37]. Therefore, PhA can be considered as a reliable prognostic marker and should be considered as a screening tool for the identification of patients at risk of deterioration of nutritional and functional status [21, 38, 39]. BIA is used as a tool to obtain data that helps to better understand the nutritional status of the patient, being a non-invasive, relatively inexpensive and easily transportable technique [38, 40]. BIA works by passing a low intensity electrical current through the body, measuring the primary components, and estimating fat mass (FM), fat free mass (FFM) and total body water. Among the BIA parameters, PhA which indirectly represents FFM, is the most clinically established as it has demonstrated a strong ability to predict outcomes in a wide variety of clinical situations [41].

PhA predicts mortality in various clinical situations [42, 43] and a potentially useful screening tool for mortality prognosis [44]. Some studies show its association with poor disease outcome, such as length of stay (LOS), mortality or need for intensive supportive therapies [43–45]. However, the use of PhA to assess pediatric patients has not been established. Further studies are needed to validate and establish recommendations for this tool in routine clinical practice.

### Normal values PhA in healthy children

Numerous studies have estimated normal PhA values in healthy pediatric populations. Several considerations should be made in this regard. De Moraes et al. [46] found gender differences in the PhA values among adolescents, with boys exhibiting significantly higher values compared to girls, even after controlling for age group and sexual maturity status. Additionally, it was found that PhA values tended to increase with advancing age and maturity. Moreover, when examining the relationship between PhA and proximity to predicted age at peak height velocity (PHV), a stronger association was observed in boys than in girls. Incorporating body mass into the multilevel models revealed that changes in overall body mass accounted for a substantial portion of the influence exerted by maturity status and age group on the PhA. These findings indicate that body size plays a significant role in shaping the relationship between PhA and developmental factors. The present study highlights the multifactorial nature of PhA variability, indicating its dependence on inter-individual differences in sex, age, maturity status, and body size. Therefore, when investigating PhA in adolescents, it is recommended to employ multilevel modeling with standardized parameters as the default approach to effectively control for the concurrent influence of sex, age, maturity status, and body size. Ballarin et al. [47] described that PhA increases progressively over the first 2 decades of life and is higher in male than female adolescents, especially after the age of 13 yrs. Less consistent evidence has been reported in younger subjects. Schmidt et al. [48]reported in a representative German sample that Percentile curves for body composition parameters are similar between boys and girls until puberty. Subsequently, girls show a higher FMI than boys, and boys increase their FFM, BCM, and PA time-shifted, in that order. Differences in FMI between the overall and the normal weight sample increase with age, showing an age-dependent prevalence for overweight and obesity.

In the study conducted by Redondo-del-Río et al. [49], tolerance ellipses were utilized to analyze the Spanish child and adolescent population. The results revealed a displacement of the mean impedance vector across various age groups, with a few exceptions. Notably, there were no significant displacements observed in girls aged 12–13 years, girls aged 15–18 years, and boys aged 16–18 years. Remarkably, the study also highlighted sex-related differences in the mean impedance vector across all age ranges, including in prepubertal children. These differences persisted throughout adolescence. The observed patterns of vector displacement were found to align with the expected timing of normal growth and development, indicating that they can be attributed to the maturation process.

Mattiello et al. [50] conducted a systematic review and meta-analysis involving 46 studies and 249,844 subjects to explore age-related variations in PhA and gender differences. The results showed that for males, the mean PhA increased from 3.6 (95% CI: 3.0–4.1) in infants (0–2 years) to 7.3 (95% CI: 7.0–7.5) in teenagers (16–18 years), stabilized in adults (18–38 years), and decreased to 5.3 (95% CI: 4.5–6.0) in elderly individuals (> 80 years). Females had similar patterns, with PhA starting at 3.7 (95% CI: 3.2–4.3) in infants, reaching 6.4 (95% CI: 6.1–6.8) in teenagers, stabilizing in adults (18–48 years), and decreasing to 5.4 (95% CI: 5.3–5.6) in elderly individuals (> 80 years). Males generally had higher PhA estimates than females, except for infants and subjects older than 80 years old.

Considering the importance of body composition analysis as a complementary nutritional assessment and as a possible predictor of morbidity and mortality in many clinical situations, studies are needed to demonstrate this ability, especially in critically ill children, because data are still scarce.

The GRADE method aims at grading the quality of evidence and grading the strength of recommendations. It has been approved to reduce the risk of bias, inconsistency of results between studies, indirect evidence, imprecision and publication bias [16, 51].

This systematic review of the literature aims to determine whether the PhA value is a good prognostic marker of morbidity and mortality and to establish the reliability of recommendations intended for use in routine clinical practice guidelines.

The PICO question asked was: In hospitalized or ambulatory PEDIATRIC patients (with disease-related malnutrition or at risk of malnutrition) does the existence of an altered PHASE ANGLE predict higher mortality and/or morbidity (short and long term)?

## Methods

### Study design

A systematic review was performed comparing studies based on the PICO (Patient, Intervention, Comparison, Outcome) framework. A MESH search was performed by applying the appropriate filters in PubMed.

The evidence was evaluated using the parameters or recommendations of the GRADE (Grading of Recommendations Assessment, Development and Evaluation) method [16], which defines the quality of evidence as the degree of confidence we have that the estimate of an effect is adequate to make a recommendation.

### Literature search

To obtain published studies related to the topic of interest, the following websites were consulted: MEDLINE or PubMed, Scopus, Embase and Web of Science (from base inception to January 2023). The following terminology was used in the title, abstract or keyword fields: ("malnutrition"[MeSH Terms] OR "malnutrition"[All Fields]) AND "phase angle"[All Fields] AND (("mortality"[Subheading] OR "mortality"[All Fields] OR "mortality"[MeSH Terms]) OR "Lenght of stay"[All Fields] OR "Quality of life"[All Fields] OR "complications"[All Fields]) AND "humans"[MeSH Terms] AND (English[lang] OR Spanish[lang]), to identify the main bioimpedance parameters along with the population of interest. In addition, the following filters were used to select the pediatric population: Filters applied: Child: birth-18 years, Infant: birth-23 months, Infant: 1–23 months, Newborn: birth-1 month, Preschool Child: 2–5 years, Child: 6–12 years, Adolescent: 13–18 years. Articles published in English or Spanish were selected for critical synthesis, and only significant associations are reported.

The clinical questions that guided the literature search were elaborated by the scientific committee with a focus on the population of interest. To determine the eligibility criteria, the PICOS strategy was adopted [52]: where "P" (patients), corresponded to pediatric patients, all genders and ethnicities; "I" (intervention), was designated as PhA bioimpedance assessment, "C" (comparison), was defined as altered versus normal PhA results, "O" (outcomes), were mortality, LOS or PICU admissions, and "S" (study design), were related to observational or clinical trials.

Exclusion criteria were as follows (i) articles did not include a full text description of the study; (ii) were not in English or Spanish; (iii) PhA differences were not assessed in relation to outcomes (e.g., mortality, length of stay, complications, or sequelae); (iv) studies published in non-peer-reviewed journals; (v) meta-analyses, reviews, protocols, case series or reports, editorials, and letters to the editor; (vi): studies on animal models.

### Risk of bias assessment

GRADE method is an approach that enables an explicit evaluation of evidence and provides a framework to develop recommendations [16] GRADE was used to evaluate the evidence regarding the prognostic value of morphofunctional assessment tools in terms of mortality and complications. For each of the seven topics, an expert reviewed the literature, selecting outcomes from the studies, rating their importance, and evaluating outcomes across studies; then the evidence profile tables for each outcome was created, including a rating of the quality of the evidence, using GRADEpro GDT software (https://gradepro.org). The tables included outcomes, number of studies, study design, risk of bias, effect, quality of evidence, and importance. Another author from the scientific committee reviewed the evidence tables and conclusions drawn from the literature. The overall quality of evidence was graded across outcomes based on the lowest quality of critical outcomes. The scientific committee then made recommendations for each topic based on the literature findings and balancing consequences (e.g., benefits/harms, values and preferences, feasibility).

### Statistical analysis

We used Review Manager 5.3 statistical software for the meta-analysis. Risk Ratio (RR) or Odds Ratio (OR) and 95% confidence interval (CI) were used in this study for continuous binary variables, respectively. it indicates that the index is statistically different between studies, and Random Effects Model (Random) is used to combine. If the heterogeneity test p > 0.05 and I2 < 50%, it indicates that there is no statistical difference in this indicator between studies, and the Fixed Effects Model (Fixed) is used to integration.

## Results

### Results in tables: evidence map and GRADE table

**Fig. 2:**
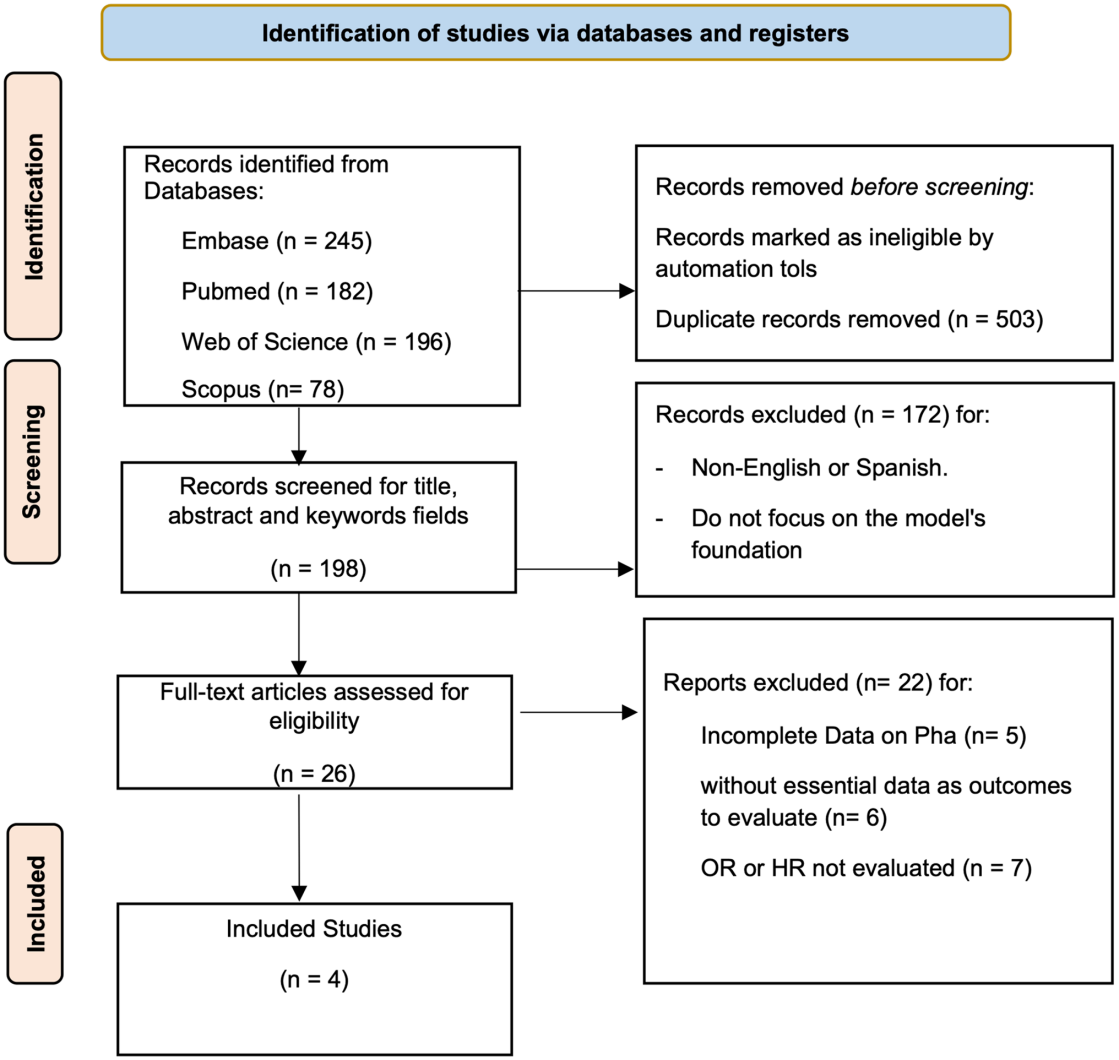
Flow-chart diagram for a systematic review of PhA and pediatric patients. PRISMA 2020

In our review we obtained a total of 701 studies, as can be seen in the Flow Chart (Fig. 2). Of these articles identified from the databases, 503 were removed before the screening process through duplication. The analysis of titles, keywords and abstracts, based on our inclusion criteria (PICOS) and exclusion criteria, identified 26 potentially eligible studies. After reading the full text, 4 relevant studies were finally included in our systematic review about PhA and pediatric patients [53–56]. 22 reports were excluded due to a lack of evaluation of PhA and poor outcomes for incomplete data in the pediatric population, without essential data or OR and HR analysis.

### Characteristics, methodology and outcomes of the included studies

Marino et al. [53] analysed the relationship between nutritional status, PhA and post-operative outcomes in children with congenital heart disease in a sample of 122 patients, aged from birth to 8 years (Table 1). They observed that lower PhA was related to worse nutritional status and longer hospital stay in PICU. ROC curves at different time points revealed that 40th percentile at day 0 PhA = 2.6° (p = 0.03), 30th percentile at day 2 PhA = 2.7° (p = 0.03) and 50th percentile PhA = 3.4° (p = 0.01), with predictive accuracy of adverse clinical outcomes at hospital discharge. Children with a PICU stay of > 4 days had significantly lower PhA value on day 0, 2 and at discharge compared to children whose PICU stay was < 4 days. These authors showed that a lower PhA was associated with worse outcome and longer hospital stay. A Pha < 2,7º on day 2 of admission leads to an increase in hospital days > 4 days, OR = 7.8 (2.7–22.45) p < 0.001 (Table 2). These are cardiac patients, many of them with elective surgery, as nowadays the age of intervention of patients with congenital heart disease has decreased considerably due to the refinement of surgical techniques and the skill of cardiac surgeons. In many cases they are operated on very early. The timing of surgery should be carefully selected and optimized according to the PhA in order to avoid the complications described above (Table 3).

**Table 1:** Characteristics and main results of the included studies:

**Variables**	**Marino et al.** [53]	**Zamberlan et al.** [54]	**Almeida de Azevedo et al.** [55]	**Xiong et al.** [56]
Publication date	28^th^ June 2017	10^th^ October 2018	April 2020	6^th^ June 2022
Country	United Kingdom	Brazil	Brazil	China
Study Design	Prospective Observational Cohort Study	Prospective Observational Study	Prospective Observational Study	Prospective Observational Study
Participants centres	One referral center	One referral center	One referral center	One referral center
Clinical profile	PICU	PICU	PICU	PICU
Patients (n, age, sex)	n = 117, 0–16 y, 61% males	n = 247, median 4,8 y (2–18 y), 129 females *(52.2%)*	n = 145, median *8.4 m (IQ 3.4–23.4),* males *(60%)*	n = 231, 1–18 y, 165 males (71,3%)
Outcomes and follow-up time	Nutrition status, PICU-LOS < 4 days, PICU LOS > 4 days and mechanical-ventilation baseline and discharge.	PICU-LOS *of up to 40 days* and *30-day mortality.*	90 days- Mortality	LOS, medical ventilation, 90-day Mortality
Event rate	PICU-LOS (> 4 days): Phase angle ≤ 2.7°: 8/28 (28,6%)	Mortality: 36/247 (14.5%)	Mortality: 5/145 (3.4%) Sepsis-shock: 24/145 (16.5%)	Malnutrition:73/231 (31.6%) Mortality: 34/231 (14.7%)
Conclusions	A PhA < 2.7° increased the odds of PICU-LOS and mechanical-ventilation.	PhA < 2.8° is significant *predictor of nutrition status, PICU-LOS and mortality.*	PhA have a potential role to support the diagnosis of septic shock.	Low PhA value is a biological marker that could be a predictor of 90‐days mortality in critically ill children. PhA < 3° could need special nutrition attention.

*PICU* Pediatric Intensive Care Unit, *LOS* Length of stay, *PhA* Phase angle, *y* years, *m* months

**Table 2:** Methodology of the included studies and bioelectrical parameters of the study groups

**Variable**	**Marino et al.** [53]	**Zamberlan et al.** [54]	**Almeida de Azevedo et al. ** [55]	**Xiong et al. **[56]
Measurement methodology (BIA-device and procedures)	ImpediMedSFB7 (Pinkenba, QLD 4008 Australia). *At 4 time* *points; with baseline before surgery, post-operative day 0, post-operative day 2, and before discharge from hospital.*	Tetrapolar body composition analyzer (model 450, Biodynamics Corporation, Seattle, WA). Anthropometric nutrition assessment was performed within 24 h of admission to characterize	BIA 101 Quantum II (RJL Systems, USA), frequency of 50 kHz. Bioelectrical impedance was measured daily until discharge or death, up to a maximum period of 14 days in the first 24 h of admission in the intensive care unit.	InBodyS10 (Biospace, Seoul, South Korea) was used for the measurements of the study.
Comparative groups	> PICU-LOS vs. < PICU-LOS	> PICU-LOS vs. < PICU-LOS. Survivors vs. Non-survivors	Survivors vs. Non-survivors. Septic shock vs free septic shock. Malnourished vs eutrophic. > LOS-hospitalization vs < LOS-hospitalization.	Survivors vs. Non-survivors.
PhA comparative groups	**Baseline:** 4.0 ± 1.1 vs. 3.8 ± 1.4, p = ns **Day 0:** 3.2 ± 1.2 vs. 2.5 ± 1.4, p = 0.005 **Day 2:** 3.8 ± 1.8 vs. 2.7 ± 0.9, p < 0.0002 **Discharge:** 3.9 ± 1.0 vs. 3.3 ± 0.7, p = 0.03	**Survival**: PhA > 2.8° vs PhA < 2,8°; the mean was 53 days (95% CI: 40.5–65.8); 23 days (95% CI: 19.7–26.8), (P < .0001). **PICU-LOS:** PhA ≤ 2.8° and PhA > 2.8° (p = 0.0013).	**Malnourished:** PhA = 2.97 vs eutrophic children PhA = 2.95, (p = 0.86). **Septic shock:** PhA were 2.61 (2.44–3.20) in those who developed septic shock any time during. Who never experienced septic shock PhA of 2.90 (2.60–3.50), p-value = 0.06. **LOS** PhA ≤ 2.61, 12 days LOS-hospitalization. PhA > 2.61 7 days LOS-hospitalization, p = 0.001	***Mortality***: 4.3° (± 1.1) vs. 3.1° (± 0.9), p < 0.05.
PhA Cut off values (AUC)	**Mechanical ventilation:** Baseline p = ns Post-op day 0 PhA 40^th^ 2.6° (AUC 0.6, 0.5–0.8), p = 0.03 Post-op day 2 PhA 30^th^ 2.7° (AUC = 0.6, 0.5–0.8), p = 0.03 Discharge from hospital PhA: 3.4° (AUC = 0.7, (0.6–0.8), p = 0.01 ***PICU-LOS*** 2.9° (AUC = 0.7,0.6–0.8), p = 0.008 2.9° (AUC = 0.6, 0.5–0.7), p < 0.04 2.7° (AUC = 0.8, 0.7–0.9), p < 0.0001 2.7° (AUC = 0.8, 0.6–1.0), p = 0.03	**Mortality**: Cut-off mortality PhA = 2.8° (AUC 0.65, 0.58–0.71), sensitivity 37.1% and specificity of 86% **PICU-LOS:** PhA ≤ 2.8° and PhA > 2.8°, (P = 0.0013)	**Predictive capacity PhA of septic shock** **At admission**: PhA = 2.78, AUC = 0.62, (0.50–0.74), sensitivity 58%, specificity 63%. OR 2.36 (0.98–5.96) **24 h before:** PhA = 3.27°,AUC = 0.62(0.58–0.67), sensitivity 95.8% and specificity 87.5% (95%CI), OR = 9.58 (1.29–71.47). **Septic-shock day:** PhA = 2.64°,AUC = 0.77(0.70–0.84), sensitivity 87.5% y specificity 67% (95%CI), OR = 14.2 (4.47–45.1).	**Phase angle 90-day mortality:** AUC 0.69 (0.53–0.85, p < 0.05), sensitivity 83%, specificity 53%, PhA = 3.0°.
OR-HR (Univariate)	**PICU-LOS (> 4DAYS)** PhA day 2 < 2.7° OR = 7.8 (2.7–22.45) p < 0.001 **Mechanical ventilation (> 2DAYS)** PhA day 0: 2.6° OR = 4.1 IC95% (1.-12.4) p = 0.01	**Mortality:** PhA, HR = 0.66 (0.48–0.89), p = 0.0073. **PICU LOS:** PhA ≤ 2.8° (HR: 1.64 (1.09–2.47) p = 0.003	**Predictive capacity PhA of septic shock** **At admission**: OR 2.36 (0.98–5.96) **24 h before:** OR = 9.58 (1.29–71.47) **Septic-shock day:** OR = 14.2 (4.47–45.1)	OR: 1.51 (1.10- 2.07) p = 0.01*

*PhA* Phase angle, *PICU* Pediatric Intensive Care Unit, *HR* hazard ratio, *OR* odds ratio, *LOS* length of stay, *AUC* area under curve

**Table 3:** Outcomes

MORTALITY	[54] Observational study of 247 children admitted aged 2–18 years. Mortality cut-off point PhA ≤ 2.8,p < 0.001 AUC = 0.65, CI 0.58–0.71. Sensitivity 37%. Effectiveness 86%. Mortality-PhA. HR = 0.66 (0.48–0.89) p = 0.0073 Days stay PICU-PhA: HR = 1.84 (1.23–2.77) p = 0.0013 PHA ≤ 2,8° higher morbidity-mortality risk and worse nutritional status.
	[56] Observational study in PICU 231 children aged 1–18 years (31.6% with malnutrition). PhA of 90-day survivors was higher than non-survivors PhA = 4.3° ( 1.1) vs. 3.1° (0.9),p = 0.02. OR = 1.51, (1.10–2.07,p = 0.01). The AUC of PhA for predicting 90-day mortality was 0.69 (0.53–0.85) p < 0.05, and the cut-off value for PhA was 3.0, with a sensitivity and specificity of 83 and 53%. A PhA < 3° was associated with a 1.51-fold increased risk of death. PhA is a predictor of mortality and longer duration of medical ventilation.
PROLONGED LENGHT HOSPITAL STAY	[56] Observational study in PICU 231 children aged 1–18 years. Days admitted to PICU by degree of malnutrition: non-malnourished: 7.00 (6.00–10.00), moderately malnourished 8.00 (3.00–11.75) and severely malnourished 11.20 (2.75–15.00), p = 0.86. A low PhA was associated with longer duration of mechanical ventilation in the PICU (r = -0,42).
	[53] Observational study. Prospective cohort study in 117 children up to 8 years of age undergoing cardiac surgery (20.6% children, 28% moderately malnourished infants). - Malnourished > PICU stay: OR 1.8 (1.1–2-7), p < 0.008 -PhA < 2.7° > PICU stay: OR 7.8 (2.7–22) p < 0.001. Measurement of PhA would help to identify patients at increased risk.
COMPLICATIONS	
UCIP-Septic shock	[55] 145 children 8.4 months on average admitted to PICU. Predictive value of Xc/h = 48.63, AUC = 0.62(0.50–0.74), OR = 3.72 (1.12–12.4). PhA = 2.78°, AUC = 0.62(0.50–0.74),OR = 2.36 (0.98–5.69) for detecting onset of septic shock. (Lower PhA and Xc were associated with higher risk of sepsis. PhA values to anticipate diagnosis: 24 h before: PhA = 3.27°, AUC = 0.62(0.58–0.67), sensitivity 95.8% and specificity 87.5% (95%CI), OR = 9.58 (1.29–71.47) Day of onset of septic shock PhA = 2.64°, AUC = 0.77(0.70–0.84), sensitivity 87.5% and specificity 67% (95%CI), OR = 14.2 (4.47–45.1). In conclusion, PhA can help in assessing the patient's risk of complication during PICU stay.
Mechanical ventilation need	[53] Observational study. Prospective cohort study in 117 children up to 8 years of age undergoing cardiac surgery (20.6% children, 28% moderately malnourished infants). - Malnourished > PICU stay: OR 1.8 (1.1–2-7), p < 0.008 -PhA < 2.7° > PICU stay: OR 7.8 (2.7–22) p < 0.001. Measurement of PhA would help to identify patients at increased risk.

*PhA* Phase angle, *PICU* Pediatric Intensive Care Unit, *OR* odds ratio, *AUC* area under curve

In an observational study by Zamberlan et al. [54] it was observed that in a sample of 247 patients (with different pathologies) aged between 2 and 18 years (Table 1), the cut-off point of PhA was 2.8° correlated with the risk of mortality, since this increased significantly in PICU hospitalized patients, showing a ROC curve analyzed under the AUC = 0.65 with a confidence interval between 0.58–0.71, sensitivity of 37% and specificity of 86% (Table 2). Thirty-six deaths occurred during hospitalization in the tertiary PICU, corresponding to 14.6% of cases. This article concluded that the lower the PhA value, the higher the risk of morbidity and mortality and the worse the nutritional status of the patient. In addition, most deaths occurred while the patient was hospitalized in PICU and a mean hospital stay of 4 days was observed, with a mortality rate 2.7 times higher, so it could also be said that the lower the PhA, the longer the hospital stay and the higher the risk of admission to PICU. This analysis was adjusted for sex and age with Cox regression and revealed that children with PhA ≤ 2.8° were more likely to stay in PICU compared to those with PA > 2.8° [HR: 1.64 (1.09–2.47); p = 0.003], (Table 3).

In a prospective analysis, Almeida de Azevedo et al. [55] conducted bioelectrical impedance measurements on 145 children aged between one month and six years who were initially not in septic shock upon admission to the PICU (Table 1). The researchers analyzed serial measurements of PhA to determine its sensitivity and specificity in accurately identifying children who later developed septic shock. The results of the study revealed that lower PhA values were associated with an increased occurrence of septic shock and longer stays in the PICU. PhA < 3.27° had an OR 9.58 (1.29–71.47) for developing septic shock the next day, with a sensitivity of 95.8%, specificity of 29.4%, and an AUC of 0.62 (0.58–0.67). Moreover, the presence of a PhA < 2.64° showed an OR of 14.2 (4.47–45.1), with an AUC of 0.77 (0.70–0.84), for developing septic shock on the same day (Tables 2 and 3).

In an observational study conducted by Xiong et al. [56], 231 pediatric patients admitted to the PICU were included, with 31.6% of them being malnourished (Table 1). The study aimed to assess the relationship between the PhA and 90-day survival. The results showed that children with a higher PhA had a longer survival time compared to those with a lower PhA (cut-off PhA = 3.0°, sensitivity 83%, specificity 53%). The area under the curve (AUC) was 0.69 (95% CI: 0.53–0.85, p < 0.05). The OR for survival with a higher PhA was 1.51 (1.10–2.07, p = 0.01) (Tables 2 and 3).

Furthermore, the study compared the duration of admission to the PICU among different degrees of malnutrition (non-malnourished, moderately malnourished, severely malnourished) and found no significant difference (p = 0.86). However, a lower PhA was associated with a longer duration of mechanical ventilation in the PICU (r = -0.42). These findings suggest that low PhA values can serve as prognostic markers for mortality risk in pediatric patients.

### Quality of studies

From the initial literature review which yielded 701 studies, only 4 articles covered all four topics related to PICOs issues. The quality of the evidence was evaluated following the GRADE method (Table 4), to make evidence-based recommendations on the prognostic and clinical value of PhA measurement (Table 5). There was insufficient evidence to make recommendations for the systematic use of PhA as an indicator of the length hospital stay. However, we could make recommendations for the usefulness of the PhA as a prognostic marker for poor outcomes such as mortality or complications in hospitalized pediatric patients. Thus, PhA, which has a weak strength of recommendation and low-very low quality of evidence, could suggest mortality and complications in hospitalized pediatric patients.

**Table 4:** GRADE evidence in Pediatric patients: Summary of the body of evidence, the judgments about the quality of the evidence, key results, and importance

**Certainty assessment**							**№ of patients**	**Effect**	**Certainty**	**Importance**
**№ of studies**	**Study design**	**Risk of bias**	**Inconsistency**	**Indirectness**	**Imprecision**	**Other considerations**	**Phase angle**	**Relative (95% CI)**		
**All-cause mortality in patients (Critically Ill Children) (follow-up: 40 days; assessed with: phase angle)**										
1 (Zamberlan et al. 2019) [54]	observational studies	not serious	not serious	not serious	not serious	none	36/247 (14.6%)	HR 0.66	⨁⨁◯◯	CRITICAL
								(0.48 to 0.89)	Low	
**All-cause mortality in patients ( Critically Ill children) (follow-up: 90 days; assessed with: phase angle)**										
1 (Xiong et al. 2022) [56]	observational studies	not serious	not serious	not serious	not serious	none	34/231 (14.7%)	OR 1.51	⨁⨁◯◯	CRITICAL
								(1.10 to 2.07)	Low	
**Prolonged length hospital stay (defined as length hospital stay > 4 day) in patients (surgery of congenital heard diseased) (assessed with: phase angle)**										
1 (Marino et al. 2018) [53]	observational studies	not serious	serious^a^	not serious	serious^d^	none	8/28 (28.6%)	OR 7.80	⨁◯◯◯	IMPORTANT
								(2.70 to 22.45)	Very low	
**Prolonged length hospital stay (defined as length hospital stay > 4 day) in patients (Critically ill children) (assessed with: phase angle)**										
1 (Zamberlan et al. 2019) [54]	observational studies	not serious	not serious	not serious	not serious^e,f^	none		HR 1.64	⨁⨁◯◯	IMPORTANT
								(1.09 to 2.47)	Low	
**Complications (defined as Mechanical ventilation need) in patients (surgery of congenital heard diseased) (assessed with: phase angle <2.6º in day 0)**										
1 (Marino et al. 2018) [53]	observational studies	not serious	serious^a^	not serious^b^	not serious^c^	none		OR 4.1	⨁⨁◯◯	IMPORTANT
								(1.0 to 12.4)	Low	
**Complications (defined as Septic shock) in patients (Critically Ill children) (assessed with: phase angle at admission <2.78º)**										
1 (Almeida de Azevedo 2020) [55]	observational studies	not serious	not serious	not serious	serious^g^	none	24/145 (16.6%)	OR 2.36	⨁◯◯◯	NOT IMPORTANT
								(0.98 to 5.96)	Very low	
**Complications (defined as Septic shock) in patients (Critically Ill children) (assessed with: phase angle 24h before de septic shock <3.27º)**										
1 (Almeida de Azevedo 2020) [55]	observational studies	not serious	not serious	not serious	serious^d^	none	24/145 (16.6%)	OR 9.58	⨁◯◯◯	IMPORTANT
								(1.29 to 71.47)	Very low	
**Complications (defined as Septic shock) in patients (Critically Ill children) (assessed with: phase angle at septic shock day <2.64º)**										
1 (Almeida de Azevedo 2020) [55]	observational studies	not serious	not serious	not serious	serious^d^	none	24/145 (16.6%)	OR 14.20	⨁⨁◯◯	IMPORTANT
								(4.47 to 45.10)	Low	

**Question**: Prognostic value of Low Phase Angle in pediatrics patients with different condition related with intensive care during short- and medium-term follow-up

**Setting: General ward and ICU admitted pediatrics patients**

*CI* confidence interval, *HR* hazard Ratio, *OR* odds ratio

*Explanations*

^a^The study population focuses on patients with heart disease pending surgery, so it cannot be extrapolated to the rest of the pediatric population

^b^Risk results associated with PICU length of stay and need for mechanical ventilation, but not mortality, are shown

^c^No sensitivity and specificity results are shown for the phase angle cut-off points measured at the different times

^d^The confidence interval of the OR has a very wide confidence interval

^e^No AUC values are shown for PICU-LOS

^f^The confidence interval of the HR has a very wide confidence interval

^g^The confidence interval intersects the value 0, so the differences are not significant

**Table 5:** Evidence-based recommendations following the GRADE method for hospitalized pediatric patients

**No.**	**Topic**	**Strength of recommendation**	**Quality of evidence**	**Recommendation**
R1	Phase angle	Weak	Very Low-Low	The phase angle, measured by bioelectrical impedance analysis, can suggest mortality in hospitalized pediatric patients.
R2	Phase angle	Weak	Very Low-Low	The phase angle, measured by bioelectrical impedance analysis, can be suggest complications (septic shock) in hospitalized pediatric patients.

### Impact of the PhA as a prognostic factor of poor outcomes in pediatric patients

**Fig. 3:**
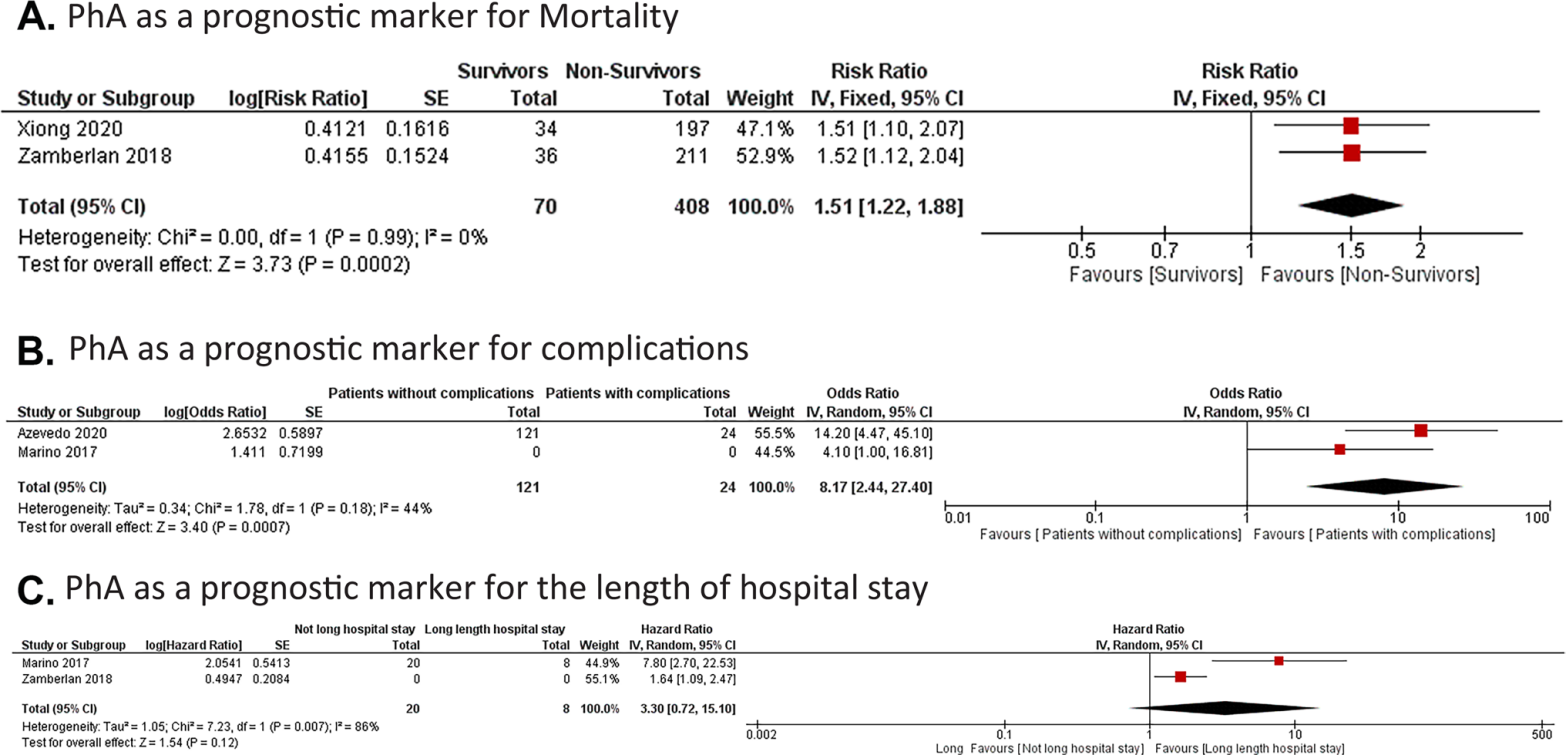
Analyses of PhA as a prognostic marker for poor outcomes in hospitalized pediatric patients. The data of OR or HR and 95% CI from 4 studies were combined in this meta-analysis and the result of the meta-analysis was described as a forest plot. Four studies were grouped, in the main poor outcomes studied: mortality (**A**), complications (**B**) and length of hospital stay (**C**). OR: Odds ratio; HR: Hazard ratio; CI: confidence interval

Finally, we examined the usefulness of the PhA as a prognostic factor for poor outcomes. We operated for meta-analysis a randomized-effect or fixed-effect model if the tests were characterized as heterogeneous or homogeneous, respectively. Meta-analysis of data from 408 patients indicated a significantly increased mortality risk in pediatric patients with lower PhA [RR: 1.51; 95% CI (1.22 – 1.88), p = 0.0002]. Homogeneity between studies: I2 = 0%, (p = 0.99). A significantly increased complications risk was found in 262 pediatric patients with lower PhA [OR: 8.17; 95% CI (2.44 – 27.4), p = 0.0007]. Heterogeneity between studies: I2 = 44%, (p = 0.18). However, PhA was not a significant predictor of longer hospital stay [RR: 3.30, 95% CI (0.72 – 15.10), p = 0.12] (Fig. 3).

## Discussion

PhA is indeed a reliable indicator of cellular health and is typically not less than 4° in a healthy pediatric population [46, 47, 49, 50]. Its measurement is simple and can provide valuable insights into the prognosis and evolution of patients. The study conducted by Marino et al. [53] further highlights the significance of PhA in assessing the prognosis of children with congenital heart disease. The results demonstrate that children who experienced longer stays in the PICU, exceeding four days, had significantly lower PhA values on day 0, day 2, and at discharge compared to those with shorter stays. This finding suggests that monitoring PhA can potentially serve as a useful tool in identifying patients at higher risk of complications and prolonged hospitalization. It is important to note that the study population of Marino et al. [53] primarily consisted of cardiac patients, many of whom underwent elective surgeries. Over time, the age at which patients with congenital heart disease undergo surgical interventions has significantly decreased due to advancements in surgical techniques and the expertise of cardiac surgeons. Consequently, surgical interventions are often performed at a very early age. Considering this, the timing of surgeries should be carefully selected and optimized based on the PhA to minimize the risk of complications associated with prolonged hospital stays and unfavorable outcomes.

The results have shown that measurements should be performed on a regular basis and can serve as a guide to predict the evolution of patients. It would be useful to have data correlating PhA values with other parameters and scores predictive of mortality in pediatric patients admitted to the PICU. This is the case for the Pediatric Index of Mortality (PIM), the Pediatric Risk of Mortality (PRISM) [57], the vasoactive-inotropic score [58], albumin or lactic acid levels whose values have not yet been correlated with the PhA [59].

The data resulting from this GRADE review with meta-analysis on the evidence of PhA as a predictor of morbidity and mortality show that a lower PhA value relates to a higher risk of complications such as sepsis and lower survival. In our systematic review was a trend for a longer hospital stay with a low PhA, but it found no statistically significant association between PhA and long length hospital stay.

It is worth remembering that in surgical pediatric patients, the intervention is not always urgent and may be postponed sometimes to find the ideal moment. In these cases, it may be advisable to optimize nutritional support and overhydration or inflammation status according to the underlying pathology by PhA monitoring.

Looking to the future, the integration of PhA measurements into routine clinical practice holds great promise. As healthcare technologies continue to advance, it is conceivable that PhA monitoring could become more accessible, allowing for frequent and non-invasive assessments of cellular health. This would enable healthcare providers to obtain real-time data on patients' inflammatory degree, as well as, nutritional-hydration status and overall well-being, facilitating timely interventions and personalized treatment plans.

Moreover, future studies could explore the potential of combining PhA measurements with other clinical parameters and predictive models to enhance risk stratification and prognostic accuracy. By incorporating PhA into existing scoring systems, such as the PIM or PRISM, healthcare professionals may gain a more comprehensive understanding of a patient's condition and make more informed decisions regarding their care [60].

As research progresses, it would be valuable to investigate the underlying mechanisms linking PhA to outcomes in pediatric patients. Consequently, understanding the physiological basis of the association between PhA and morbidity/mortality could provide insights into the complex interplay between cellular health, immune function, and disease progression in this population. This knowledge may open avenues for personalized and targeted interventions and therapeutic strategies aimed at improving outcomes and reducing complications [61].

Lastly, interdisciplinary collaborations among clinicians, researchers, and technologists will be vital in advancing the field of PhA monitoring. By combining expertise from various domains, innovative approaches can be developed to refine PhA measurement techniques, establish standardized protocols, and harness the potential of machine learning and artificial intelligence to extract meaningful patterns and predictive algorithms from PhA data.

In summary, the future of PhA as a predictor of poor outcomes and mortality in pediatric patients is promising. With continued research and technological advancements, PhA monitoring has the potential to revolutionize pediatric healthcare by enabling early detection of clinical deterioration, optimizing and personalizing treatment strategies, and ultimately improving patient outcomes. This new tool offers important opportunities to enhance the quality of care and promote better health outcomes for the children population in the years to come.

## Data Availability

The data that support the findings of this study are available from the corresponding author upon reasonable request.

## Abbreviations

BI: Bioelectrical impedance
CI: Confidence interval
ECW: Extracellular water
FFM: Fat-free mass
GRADE: Grading of Recommendations Assessment, Development and Evaluation
HR: Hazard ratio
PICU: Pediatric intensive care unit
IMV: Intensive Mechanical ventilation
LOS: Length of stay
OR: Odds ratio
HR: Hazard ratio
PhA: Phase angle
PICOS: Patient, Intervention, Comparison, Outcome, Studies
PRISMA: Preferred Reporting Items for Systematic Reviews
R: Resistance
Xc: Reactance
RR: Risk Ratio
AUC: Under the curve
PICOS: Patient, Intervention, Comparison, Outcome
